# Carbonic anhydrase 2 (CAII) supports tumor blood endothelial cell survival under lactic acidosis in the tumor microenvironment

**DOI:** 10.1186/s12964-019-0478-4

**Published:** 2019-12-17

**Authors:** Dorcas A. Annan, Nako Maishi, Tomoyoshi Soga, Randa Dawood, Cong Li, Hiroshi Kikuchi, Takayuki Hojo, Masahiro Morimoto, Tetsuya Kitamura, Mohammad Towfik Alam, Kazuyuki Minowa, Nobuo Shinohara, Jin-Min Nam, Yasuhiro Hida, Kyoko Hida

**Affiliations:** 1 Department of Vascular Biology and Molecular Pathology, Hokkaido University Graduate School of Dental Medicine, N13 W7, 060 8586, Kita-ku, Sapporo, 060-8586 Japan; 2 Institute for Advanced Biosciences, Keio University, Tsuruoka, Yamagata, 997-0035 Japan; 3 Department of Renal and Genitourinary Surgery, Graduate School of Medicine, Hokkaido University, N15 W7, Kita-ku, Sapporo, Japan; 4 Department of Dental Anesthesiology, Hokkaido University Graduate School of Dental Medicine, N13 W7, 060 8586, Kita-ku, Sapporo, Japan; 5 Department of Dental Radiology, Hokkaido University Graduate School of Dental Medicine, N13 W7, 060 8586, Kita-ku, Sapporo, Japan; 6 Global Institution for Collaborative Research and Education (GI-CoRE), Faculty of Medicine, Hokkaido University, N15 W7, 060-8638, Kita-ku, Sapporo, Japan; 7 Department of Cardiovascular Thoracic Surgery, Hokkaido University Graduate School of Medicine, N15 W7, 060-8648, Kita-ku, Sapporo, Japan

**Keywords:** Lactic acidosis, Tumor endothelial cells, Carbonic anhydrase 2 (CAII), pH regulation, Angiogenesis

## Abstract

**Background:**

Tumor endothelial cells (TECs) perform tumor angiogenesis, which is essential for tumor growth and metastasis. Tumor cells produce large amounts of lactic acid from glycolysis; however, the mechanism underlying the survival of TECs to enable tumor angiogenesis under high lactic acid conditions in tumors remains poorly understood.

**Methodology:**

The metabolomes of TECs and normal endothelial cells (NECs) were analyzed by capillary electrophoresis time-of-flight mass spectrometry. The expressions of pH regulators in TECs and NECs were determined by quantitative reverse transcription-PCR. Cell proliferation was measured by the MTS assay. Western blotting and ELISA were used to validate monocarboxylate transporter 1 and carbonic anhydrase 2 (CAII) protein expression within the cells, respectively. Human tumor xenograft models were used to access the effect of CA inhibition on tumor angiogenesis. Immunohistochemical staining was used to observe CAII expression, quantify tumor microvasculature, microvessel pericyte coverage, and hypoxia.

**Results:**

The present study shows that, unlike NECs, TECs proliferate in lactic acidic. TECs showed an upregulated CAII expression both in vitro and in vivo. CAII knockdown decreased TEC survival under lactic acidosis and nutrient-replete conditions. Vascular endothelial growth factor A and vascular endothelial growth factor receptor signaling induced CAII expression in NECs. CAII inhibition with acetazolamide minimally reduced tumor angiogenesis in vivo. However, matured blood vessel number increased after acetazolamide treatment, similar to bevacizumab treatment. Additionally, acetazolamide-treated mice showed decreased lung metastasis.

**Conclusion:**

These findings suggest that due to their effect on blood vessel maturity, pH regulators like CAII are promising targets of antiangiogenic therapy.

Video Abstract

**Graphical abstract:**

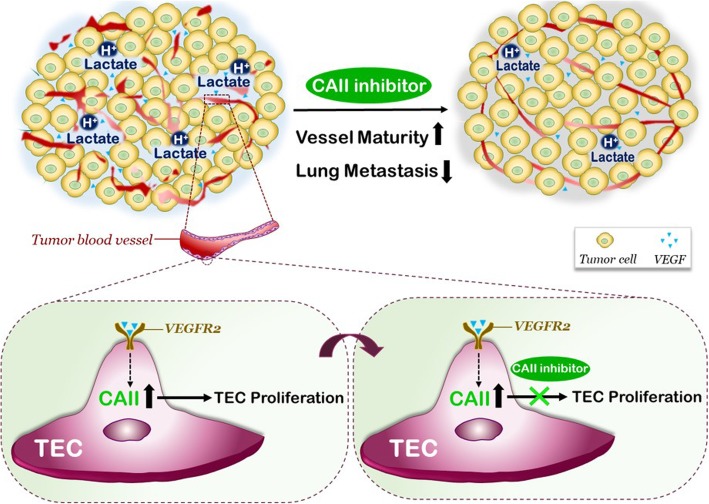

## Background

Tumor development and survival depend on angiogenesis [1]. Tumor blood vessels harbor tumor endothelial cells (TECs), which differ from NECs due to altered genetics [2] and function [3]. Alterations of TECs are caused by epigenetic changes [4, 5], influence from tumor-derived factors [6], tumor hypoxia [7], and reactive oxygen species [8]. In addition, tumor metabolic products may also influence the function of endothelial cells in the tumor microenvironment. Tumor cells preferentially undergo glycolysis in the presence of oxygen, a process known as the Warburg effect, which yields lactic acid instead of pyruvate as the end product [9]. Lactic acid exerts several effects on immune cells: it stimulates tumor-associated macrophages toward an M2-like phenotype [10] and inhibits proliferation of human cytotoxic T lymphocytes and cytokine production [11]. Although the effects of acidity [12] and lactate [13] on NECs, have been independently reported, their cumulative effect (i.e., a state of *lactic acidosis*) on TECs, remains unknown. TECs were recently described as hyperglycolytic [14], implying that they are a potential source of lactic acid in the tumor microenvironment. Consequently, in addition to tumor-derived lactic acid, TECs may also be exposed to their lactic acid. Here, we hypothesized that TECs possess unique properties that promote their survival in a lactic acid-rich environment. To address this hypothesis, we identified the response of TECs and NECs to lactic acidosis., as well as the possible mechanism involved in their survival under lactic acidosis.

Cancer cells have devised several ways to escape the harmful effects of lactic acid. They show the upregulated expression of proton-coupled monocarboxylate transporters (MCTs), such as MCT1 and MCT4, to help regulate intracellular lactate and proton levels [15]. Additionally, pH regulators including sodium/hydrogen exchanger 1 (NHE1), proton-sensing G-protein-coupled receptors, vacuolar ATPases, and carbonic anhydrases (CAs), including CAIX, CAXII, and CAII, are upregulated to maintain a stable intracellular acid-base balance [16]. Unlike other CAs, cytosolic CAII is reportedly expressed in the endothelium of melanomas and esophageal, renal, lung [17], and brain cancers [18]. Also, endothelial CAII expression has been associated with the malignant progression of meningiomas [19]. Such findings suggest that CAII plays a significant role in either the establishment of tumor endothelium or the biological activity of TECs and that tumor cells are involved in endothelial CAII expression in the tumor microenvironment. However, the unique function of endothelial CAII in the tumor blood vessels or TECs has not been elucidated. Furthermore, apart from the role of MCT1 in lactate transport, inhibition of endothelial lactate uptake via MCT1 impedes tumor angiogenesis [20], suggesting that pH regulators can influence tumor vessel growth. Here we demonstrate the involvement of CAII in the survival and proliferation of TECs.

Using primary cultured endothelial cells, we show that tumor-derived factor VEGFA can induce the expression of CAII in endothelial cells. CAII targeting affected the growth of endothelial cells in vitro under various metabolic substrates. Furthermore, pharmacological inhibition of CAs enhanced blood vessel maturation and decreased tumor metastasis in vivo.

## Materials and methods

### Mice

Six-week-old female nude mice (BALB/c Ajc1-nu/nu) were purchased from CLEA, Tokyo, Japan, and housed under specific pathogen-free conditions. All animal care and experimentation procedures adhered to institutional guidelines and were approved by the animal research authorities of Hokkaido University.

### Cells and cell culture

Highly metastatic human A375-SM melanoma cells were kindly provided by Dr. Fidler (M.D. Anderson Cancer Center, Houston, TX, USA). A375-SM cells were cultured in Minimum Essential Medium (MEM; Gibco, Thermo Fisher Scientific, Waltham, MA, USA) supplemented with 10% heat-inactivated fetal bovine serum (FBS) and 1% penicillin/streptomycin antibiotics (Sigma-Aldrich, St. Louis, MO, USA). HMVECs were obtained from Lonza (Tokyo, Japan) and used between the 4th and 8th passages. MS1 (MILE SVEN 1) murine endothelial cells were cultured in Dulbecco’s Minimum Essential Medium (DMEM; Gibco, Thermo Fisher Scientific, Waltham, MA, USA) supplemented with 10% heat-inactivated FBS and 1% penicillin/streptomycin antibiotics. All cells were free of mycoplasma contamination. The pH of the extracellular culture medium was measured using a pH electrode (Mettler Toledo).

### Human tissue samples

Tumor tissues were surgically excised from clinically diagnosed renal cell carcinoma (RCC) patients. Each patient’s healthy renal tissues were separated from the tumor tissues for comparison. All protocols were approved by the Institutional Ethics Committee of Hokkaido University, and all patients provided written informed consent before surgery. The study was performed according to the ethical guidelines of the Declaration of Helsinki.

### Isolation of TECs and NECs

A375-SM tumor cells were subcutaneously injected into the right flanks of female nude mice. TECs and NECs were isolated from the tumor xenografts and the dermis of tumor-free counterparts, respectively. ECs were isolated using CD31 microbeads and a magnetic cell sorting device (Miltenyi Biotec, Bergisch Gladbach, Germany), followed by flow cytometry (FACS, Aria II; BD Biosciences, San Jose, CA, USA) with CD31 and CD45 antibodies. CD31^+^ cells were cultured in EGM-2 MV (Lonza) containing 15% FBS at 37 °C in a humidified atmosphere with 5% CO_2_. The remaining human tumor cells carrying the DT receptor were eliminated using diphtheria toxin (DT, 500 ng/mL; Calbiochem, San Diego, CA, USA). Isolated ECs were further purified using FITC-BS1-B4 lectin (Vector Laboratories, Burlingame, CA, USA). TECs and NECs were determined to be positive for EC markers (Pecam1, Eng, Cdh5, Icam1, Flt1, and Kdr) and negative for hematopoietic markers (Itgam and Ptprc). cDNA from MS1 cells was used as a control for EC markers, and the cDNA from CD31-negative cells as a control for non-EC markers. All antibodies used are listed in Additional file 1: Table S1.

### Metabolomic analysis

TECs and NECs were seeded at 1 × 10^6^ cells density and cultured overnight. The old medium was aspirated, and the cells were washed with 5% mannitol (Wako, Osaka, Japan). Metabolites were extracted with methanol containing 25 μM each of L-methionine sulfone (Alfa Aesar, Heysham, UK), MES (Dojindo, Kumamoto, Japan), and CSA (Wako) at 10,000 g for 3 min at 4 °C. The aqueous layer was collected and concentrated at 9100 g for 2 h at 20 °C. The centrifugal concentrate was stored at − 80 °C until analysis. The concentrates were diluted with water containing 3-aminopyrrolidine (Sigma-Aldrich) and trimesate (Wako). The metabolite analyses were performed by capillary electrophoresis time-of-flight mass spectrometry (CE-TOFMS) as previously described [21].

### Lactate measurement

Lactate levels in cell culture supernatants and mouse tissues were enzymatically determined using an L-Lactate Assay Kit (Abcam, Cambridge, UK), according to the manufacturer’s instructions.

### In vitro cell proliferation assays with sodium lactate and lactic acid

NECs and TECs were cultured in nutrient-free DMEM (Sigma-Aldrich), supplemented with 10% FBS and 100 μg/mL endothelial cell growth supplement (ECGS, Corning, Discovery Lab Ware Inc., Bedford, MA, USA), containing sodium lactate or lactic acid (Sigma-Aldrich). Cell proliferation was measured using the MTS assay. All pH measurements were performed immediately before the media was added to the cells. All experiments were performed in triplicate.

### Lactic acidosis and lactosis

Lactic acidosis/lactosis conditions were created by adding sodium lactate (Sigma-Aldrich) to DMEM (Sigma-Aldrich) containing 10% FBS, 20 mM HEPES (Sigma-Aldrich), and 100 μg/mL ECGS (Corning) to a final concentration of 20 mM. The medium’s pH was adjusted to 6.9 and 7.3 for lactic acidosis and lactosis, respectively, using NaOH (Sigma-Aldrich).

### In vitro cell proliferation assay (post-small interfering RNA (siRNA) transfection)

Cells were treated with 20 nM siRNA and seeded at 2 × 10^3^ cells density in transfection medium directly into 96-well plates. After 6 h, the medium was changed to complete medium, ECGM-MV2 (PromoCell), which is fully supplemented with glucose and glutamine, thus creating a nutrient-replete environment. The cell proliferation was measured after 72 h, using the MTS assay.

In some cases, after 24 h, the medium was changed to either lactic acidosis or lactosis.

### Isolation of RNA and real-time quantitative reverse-transcription PCR (RT-qPCR)

Total RNA was isolated using the ReliaPrep™ RNA Cell Miniprep System (Promega Corporation, Madison, WI, USA), according to the manufacturer’s instructions. The cDNA was synthesized using ReverTra-Plus (Toyobo Co., Japan). Real-time RT-qPCR was performed using the KAPA SYBR® Fast qPCR Kit (KAPA Biosystems Pty (Ltd.), Cape Town, South Africa). The cycling conditions were set according to the CFX manager (Bio-Rad, Hercules, VA, USA). The expression of all messenger RNAs (mRNAs) was normalized to those of Rps13 for murine cells and RSP13 for human cells. The PCR products were observed on 2% agarose gels (Promega) and DNA stained with MIDORI ^Green^ Xtra nucleic acid gel stain (Nippon Genetics Europe GmbH, Düren, Germany). The primers used are listed in Additional file 2: Table S2.

### Knockdown by siRNA

Specific siRNAs targeting MCT1, and CAII mRNAs were introduced into TECs using Lipofectamine RNAiMAX Transfection Reagent (Invitrogen, Carlsbad, CA, USA). A nontargeting control siRNA (Invitrogen, Carlsbad, CA, USA) was used as a negative control. The siRNAs targeted the following sequences:

- *Mct1 si* 5′-GGCUUGAUCGCAGCUUCUUUCUGUA-3′,
- 5′-UACAGAAAGAAGCUGCGAUCAAGCC-3′;
- *Car2 si* 5′-CCAUUACUGUCAGCAGCGAGCAGAU-3′
- 5′-AUCUGCUCGCUGCUGACAGUAAUGG-3′

### Immunohistochemistry (IHC)

Frozen sections of A375-SM tumors were prepared as previously described [3]. Immunofluorescence analysis was performed by double staining with anti-CAII and anti-CD31 antibodies. Secondary antibodies conjugated to Alexa fluor 488 and 647 fluorochromes were used for detection followed by counterstaining with DAPI. The images were obtained with the FV10i 2.1 Viewer Software at room temperature, with a camera coupled to an objective lens with × 2.0 confocal aperture (Olympus). The Olympus FluoView ver.4.2. b software was used for image processing.

Serial sections were obtained from FFPE blocks of human RCC tumor and its normal counterparts. The sections were individually stained with anti-CAII and anti-CD31 antibodies. Immunoreactivity was visualized with HRP-linked secondary antibody (Dako) and counterstained with hematoxylin (Wako). For vessel maturity analysis, determined by the microvessel pericyte coverage index (MPI), mouse tumor FFPE sections were systematically co-stained with both anti-CD31 and anti-α-SMA antibodies in the same tissue. The anti-glut1 antibody was used to identify hypoxic tumor areas. Images were captured using a NanoZoomer 2.0-HT Slide Scanner (NanoZoomer 2.0 HT, version 2.3.27, Hamamatsu, Japan) and observed with the NanoZoomer Digital Pathology software. The antibodies used are listed in Additional file 1: Table S1.

### Evaluation of microvessel density (MVD) and microvessel pericyte coverage index (MPI)

Microvessel density was determined by selecting five hotspots (blood vessel-rich areas) and measuring the CD31-positive area. The MPI was calculated as the percentage of CD31-positive vessels associated with α-SMA-positive cells to the total number of microvessels in each hotspot.

### Western blotting

Cells were lysed using RIPA buffer (Cell Signaling Technology) alone for total protein collection and RIPA buffer with 10% SDS for membrane protein. The total protein concentration was determined using the BCA Protein Assay Kit (Pierce, Rockford, IL, USA). The western blotting procedure was performed according to a standard protocol with an antibody against MCT1, as previously described [22].

### Determination of CAII by enzyme-linked immunosorbent assay (ELISA)

CAII protein levels in NECs and TECs were determined using an ELISA kit (Novus Biologicals, USA), according to the manufacturer’s instructions. The concentration was normalized to the cell number.

### Tumor-conditioned medium (CM) preparation and heat inactivation

A375-SM tumor cells were seeded at a 1 × 10^6^ cells density in 10% FBS MEM for 48 h. Tumor-CM was collected from A375-SM cultures and filtered with a 0.22 μm filter (Millipore). The CM was mixed with equal portions of 5% FBS EBM2. The CM from the HMVEC culture was similarly prepared and used as control.

Conditioned media (CM) heat inactivation was performed at 95 °C for 60 min. After cooling down, the hNECs were treated with CM for 24 h. RNA was isolated, and CAII expression was determined by RT-qPCR.

### VEGF stimulation and VEGFR kinase inhibitor assay

hNECs were seeded and serum-starved for 24 h. The cells were stimulated with 20 ng/mL recombinant human VEGF_165_ (PeproTech, Rocky Hill, NJ, USA) for 24 h. VEGF signaling was inhibited using either bevacizumab (VEGF neutralizing antibody; Chugai Pharmaceutical Co., Japan) or the VEGFR2 kinase inhibitor Ki8751 (Calbiochem, UK). For Ki8751 treatment, cells were pretreated with 10 μM Ki8751 for 2 h, followed by treatment with Ki8751-containing medium for 24 h. CAII mRNA and protein expressions were determined by RT-qPCR and western blotting, respectively.

### In vivo pharmacological inhibition of angiogenesis with acetazolamide and bevacizumab

TdTomato Luc-2-expressing A375-SM tumor cells (1 × 10^6^) were subcutaneously implanted into the right flanks of female nude mice. After 9 days, when the tumors were visible, the mice were divided into control, acetazolamide, and bevacizumab groups of five mice each. Acetazolamide and bevacizumab were intraperitoneally administered at 40 mg/kg/daily and 5 mg/kg/every third day, respectively. Tumor volumes were measured periodically. After 27 days of treatment, the tumors were excised, and lung metastases were observed by bioluminescence imaging using an IVIS Spectrum (Caliper Life Sciences).

### Statistical analysis

Unless otherwise stated, all data are presented as mean ± standard deviation. A two-way Student’s *t*-test was used for comparison between two groups. A *P*-value < 0.05 was considered significant, and a *P*-value < 0.0001 was considered very significant.

## Results

### Glycolysis activation is higher in TECs than in NECs

**Fig. 1:**
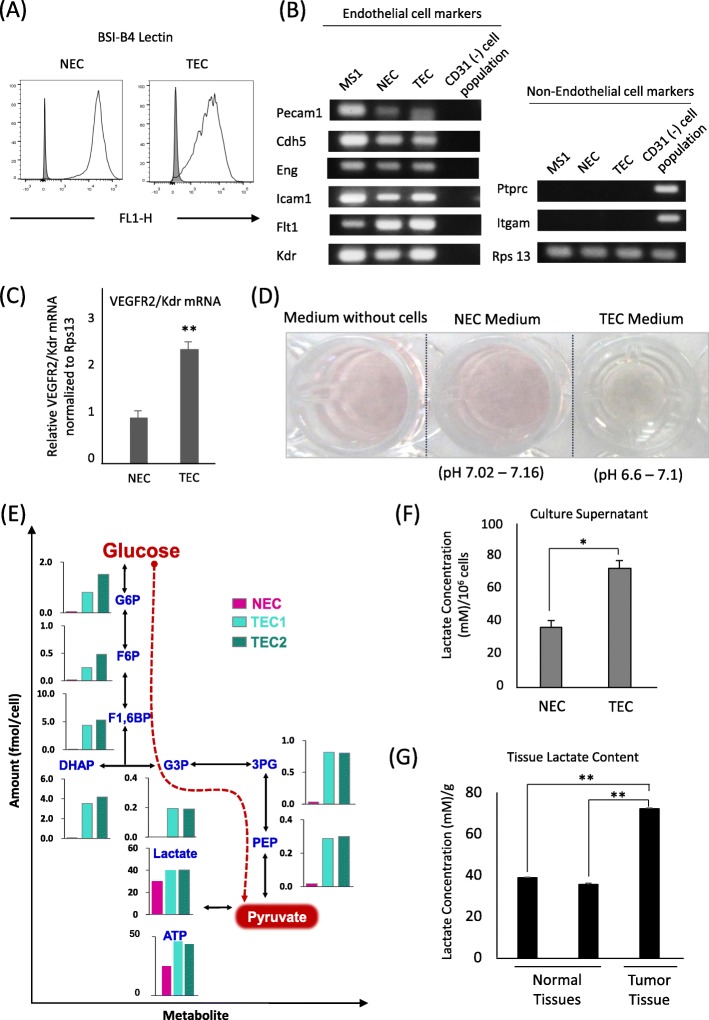
Glycolysis activation is higher in TECs than in NECs. **a** ECs’ flow cytometry analysis showing BSI-B4 lectin’s expression in stained (white) and unstained cells (gray). **b** Positive expression of EC markers (Pecam1, Cdh5, Eng, Icam1, Flt1, and Kdr). The murine endothelial MS1 cells cDNA was used as a positive control sample for EC markers. All ECs were negative for non-EC markers Itgam and Ptprc. Gene expression was analyzed by RT-qPCR, and PCR products were visualized by gel electrophoresis. **c** Kdr/VEGFR2 mRNA expression in TECs and NECs was evaluated by RT-qPCR. All results presented as mean ± SD; *n* = 3, **P < 0.001, ***P < 0.0001, by two-tailed unpaired Student’s *t*-test. **d** Images of the media with no cells and of the spent medium of TEC and NEC cultures after cells reached confluence; the extracellular pH of media was measured with a pH probe. **e** Comparison of the metabolomes of NECs and TECs cultured in complete medium (i.e., containing glucose, glutamine, and growth factors). **f** Lactate levels determined enzymatically in the media of cells cultured for 48 h. The lactate concentration was normalized to cell number. **g** Enzymatically determined lactate levels in supernatants of minced skin, kidney, and tumor tissues prepared immediately after resection. Lactate concentration was normalized to tissue weight (g). Results presented as mean ± SD; The experiment was independently repeated three times; *P < 0.05, **P < 0.001, by two-tailed unpaired Student’s *t*-test

We isolated TECs and NECs from xenografted tumors and the dermis of tumor-free mice, respectively. Prior to experiments, cells were characterized. Both TECs and NECs expressed known endothelial cell markers, such as BS1-B4 (Fig. 1a), Pecam1, Eng, Cdh5, Icam1, Flt1, and Kdr (Fig. 1b). All ECs were negative for the hematopoietic markers Itgam and Ptprc (Fig. 1b). Compared with NECs, TECs exhibited upregulated Kdr expression (Fig. 1c). The expression of EC markers indicated high purity of isolated ECs.

During the culture of NECs and TECs in media containing a pH indicator, the TEC culture medium turns pale at an earlier time point than the NEC medium. Cultures were prepared for each cell type with twice the number of TECs for NECs to compensate for the higher proliferative rates of TECs. The pH values of the culture supernatants were measured and found to be within the ranges of 6.6–7.1 and 7.02–7.16 for TECs and NECs, respectively (Fig. 1d), indicating that TECs released protons into the media and were exposed to an acidic environment. Interestingly, however, their proliferation was uninhibited by the media’s pH decrease. Considering the relationship between extracellular acidification and metabolism, we measured the metabolomes of ECs to verify the contribution of cellular metabolism to this rapid change in the media’s pH. The results revealed that TECs had a more glycolytic metabolome than NECs, which was characterized by higher levels of glycolytic metabolites and lactate (Fig. 1e). We confirmed that TECs released more lactate into culture medium than NECs (Fig. 1f). Consistently, whole-tumor lactate level was comparatively higher than in normal skin and kidney tissues (Fig. 1g), further confirming that TECs are exposed to a lactate-rich environment in vivo. These results indicate the ability of TECs to survive in a high lactate environment, which may also be acidic, due to tumor or TEC glycolytic activity.

### TECs proliferate in lactic acidosis

**Fig. 2:**
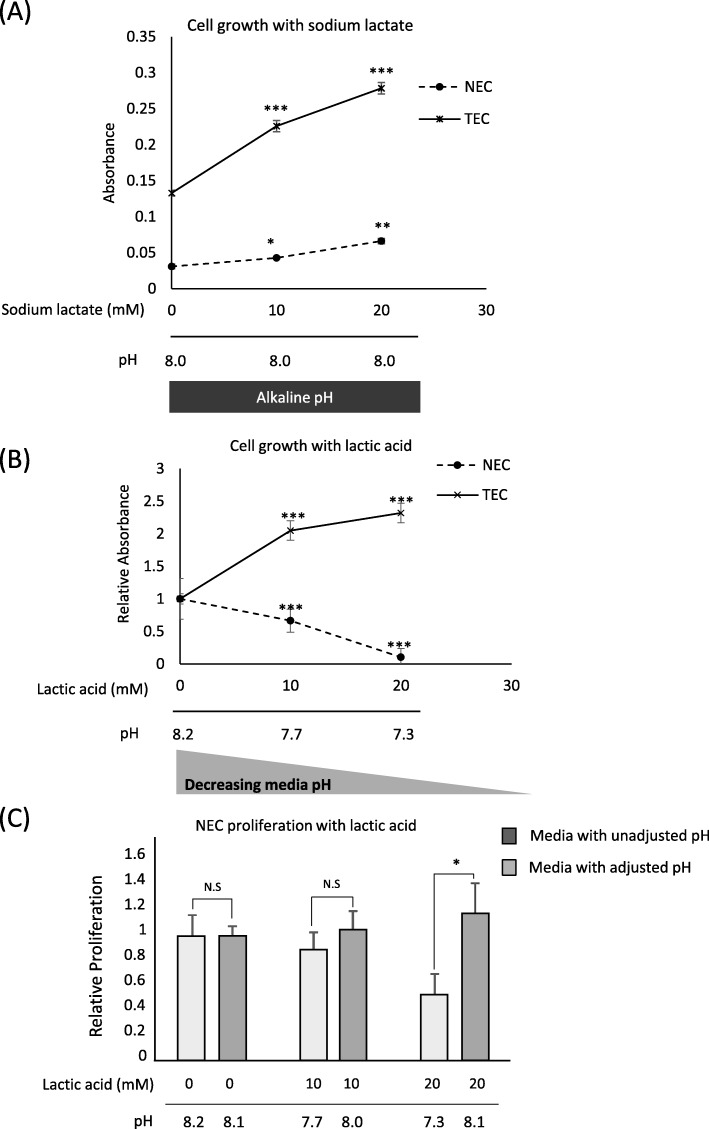
TECs proliferate in low pH media in the presence of lactate. **a** TEC and NEC proliferation in sodium lactate-supplemented medium at indicated concentrations; the media pH (alkaline pH) was measured immediately before plates were placed in the incubator; cell proliferation was measured using the MTS assay after 72 h. Data represented as absorbances (**b**) TEC and NEC proliferation in lactic acid-supplemented medium at indicated concentrations; the media pH (decreasing pH) was measured immediately before plates were placed in the incubator; cell proliferation was measured using the MTS assay after 72 h. Data represented as fold change relative to 0 mM condition. **c** NEC proliferation in pH-adjusted, 20 mM lactic acid-supplemented medium; the media pH was adjusted to approximately 8 before application to the cells; cell proliferation was measured by the MTS assay after 72 h. Data represented as fold change relative to cells in media without pH adjustment. Results presented as mean ± SD. The experiment was independently repeated at least three times; *P < 0.05, **P < 0.001, ***P < 0.0001, by two-tailed unpaired Student’s *t*-test; N.S., not significant

Previous studies involving endothelial cells focused on either the effect of lactate [13] or acidity [12, 23] on angiogenesis. First, we demonstrated that lactate could support endothelial cell growth, evidenced by a dose-dependent increase in proliferation by both TECs and NECs with sodium lactate (Fig. 2a). This increase was higher in TECs than in the NECs. Secondly, we investigated the cumulative effect of high lactate and low pH on endothelial cell proliferation by exposing ECs to increasing doses of lactic acid. TECs proliferated in a dose-dependent manner, whereas NEC proliferation was inhibited (Fig. 2b). Upon noting that the medium’s starting pH increased with the lactic acid concentration, we investigated the effect of exposing NECs to a low pH medium as opposed to a higher pH at cell culture onset. After increasing cell culture onset pH to approximately 8, NEC proliferation in 20 mM lactic acid increased (Fig. 2c), which was not previously observed (Fig. 2b). TECs can withstand slight decreases in extracellular pH and are therefore better adapted to proliferate under low pH conditions in the presence of lactate.

### Upregulated cytosolic carbonic anhydrases in TECs

**Fig. 3:**
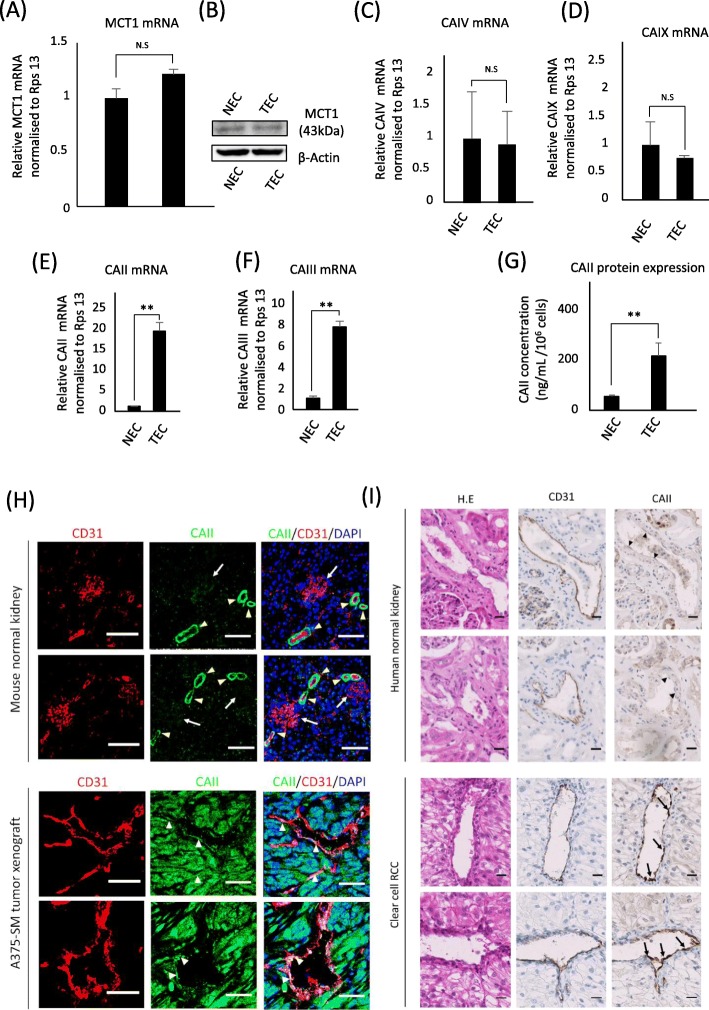
Upregulated cytosolic carbonic anhydrases in TECs. **a** MCT1 mRNA expression in TECs and NECs was evaluated by RT-qPCR. **b** MCT1 protein levels in NECs and TECs analyzed by western blotting. **c**, **d**, **e**, **f** mRNA expression of the pH regulators CAIV, CAIX, CAII, and CAIII evaluated by RT-qPCR in TECs and NECs. **g** Cellular levels of CAII in NECs and TECs were confirmed by ELISA. **h** Double immunofluorescence staining for CD31 (red) and CAII (green) in normal mouse tissue (kidney) and A375-SM tumor xenografts. White arrows point to CAII-negative glomerulus and yellow arrowheads to CAII-positive tubules. White arrowheads indicate CAII-positive blood vessels in the A375-SM tumor. Merged image shows the localization of CAII (green) and CD31 (red), and DAPI (blue) Scale bar, 50 μm. **i** CAII expression in CD31-positive blood vessels in human RCCs (arrows) and absence in normal renal tissue blood vessels (arrowheads). Scale bar, 50 μm. Results presented as mean ± SD, **P < 0.001, by two-tailed unpaired Student’s *t*-test; N.S., not significant, *n* = 3)

Monocarboxylate transporters (MCTs) are known for their unique lactate shuttling role in normal [24] and transformed [25] cells, which facilitates the adaptation of tumor cells to variations in metabolic substrate availability [25]. Here, we analyzed the expression of MCT1, which is the main MCT involved in lactate-induced angiogenesis in ECs [13]. Consistent with this report, MCT1 expression was comparable in both NECs and TECs at the mRNA and protein levels (Fig. 3a and b). Since the association of MCTs with certain pH regulating CAs promotes MCT transport activity [26, 27], we analyzed CAs in the ECs. mRNA expression analysis showed comparable expression of extracellular CAIX (Fig. 3c) and membrane-bound CAIV (Fig. 3d) in NECs and TECs. However, unlike NECs, TECs exhibited upregulated expression of cytosolic CAII (Fig. 3e) and CAIII (Fig. 3f). NECs and TECs had similar expression levels for other cancer-associated pH regulators analyzed (Additional file 3: Figure S1). Further work has focused on the role of CAII in TECs, due to its high catalytic activity [24] and its role in tumor malignancy [28, 29]. Subsequently, TECs showed a higher CAII protein level than NECs (Fig. 3g). CAII expression was confirmed in vivo, and normal murine kidney tissues were stained to validate the CAII antibody reactivity. As expected, CAII was stained in the kidney tubules, and absent in the glomeruli [17] (Fig. 3h). In A375-SM tumors, distinct CAII expression was observed in the cytoplasm of both blood endothelial cells and tumor cells (Fig. 3h). Furthermore, CAII was uniquely expressed in human renal cancer endothelium, but not in the blood vessels of the normal kidney (Fig. 3i). These observations are consistent with those of previous reports [17, 18] associating CAII with the tumor endothelium, which we have now shown to be the case in isolated TECs.

### TECs are more sensitive to carbonic anhydrase II inhibition

**Fig. 4:**
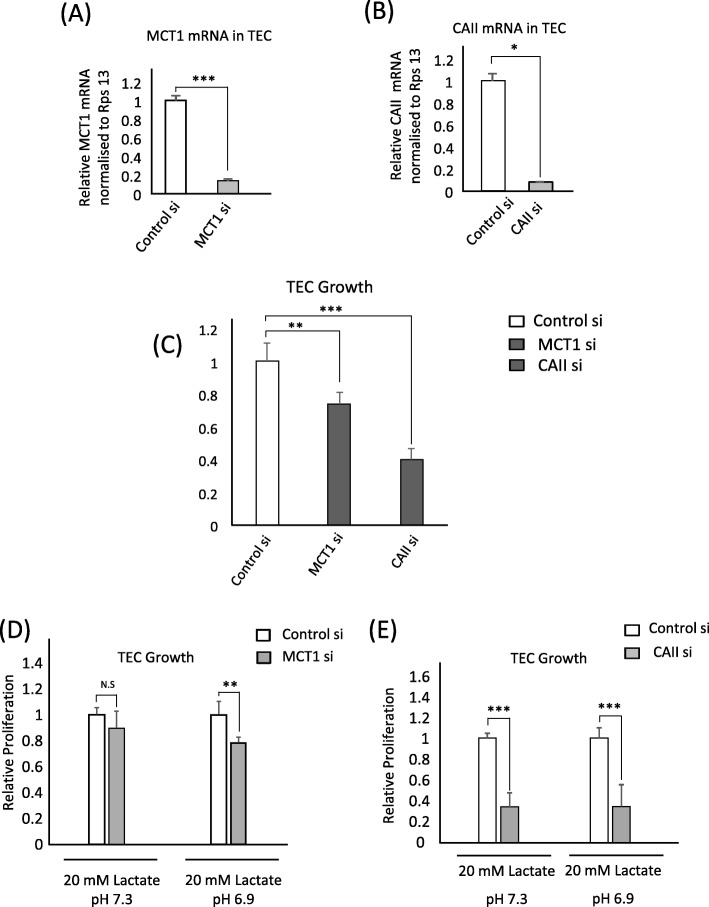
TECs are more sensitive to carbonic anhydrase II inhibition. **a**, **b** MCT1 and CAII inhibition by siRNA knockdown in TECs was confirmed by RT-qPCR. **c** The proliferation of MCT1 and CAII knockdown TECs in complete medium measured by the MTS assay. The values refer to fold change relative to control siRNA-transfected cells. **d**, **e** MCT1 and CAII knockdown TECs were exposed to lactosis (20 mM lactate, pH 7.3) and lactic acidosis (20 mM lactate, pH 6.9), 24 h after knockdown. Cell proliferation was determined by the MTS assay. The values refer to the fold change relative to control siRNA-transfected cells. Results presented as mean ± SD, *P < 0.01, **P < 0.001; N.S., not significant, *n* = 3

Considering that MCT1 inhibition can decrease tumor angiogenesis in vivo [20] and the upregulated CAII expression in TECs, we addressed the functional roles of MCT1 and CAII in TECs. Using siRNA to successfully knock down MCT1 and CAII (Fig. 4a, b), we observed that, in fully supplemented culture media (complete medium), MCT1 knockdown expectedly decreased TEC proliferation. Moreover, inhibiting CAII led to a more pronounced decrease in TEC proliferation than inhibiting MCT1 (Fig. 4c). When the media was changed to lactic acidosis and lactosis, MCT1 inhibition decreased TEC proliferation only in lactic acidosis (Fig. 4d), whereas CAII knockdown decreased TEC proliferation in both lactic acidosis and lactosis (Fig. 4e). These results suggest that CAII is generally involved in EC proliferation regardless of nutrient availability or the metabolic substrate in play.

### Tumor-derived factors induce CAII upregulation in endothelial cells

**Fig. 5:**
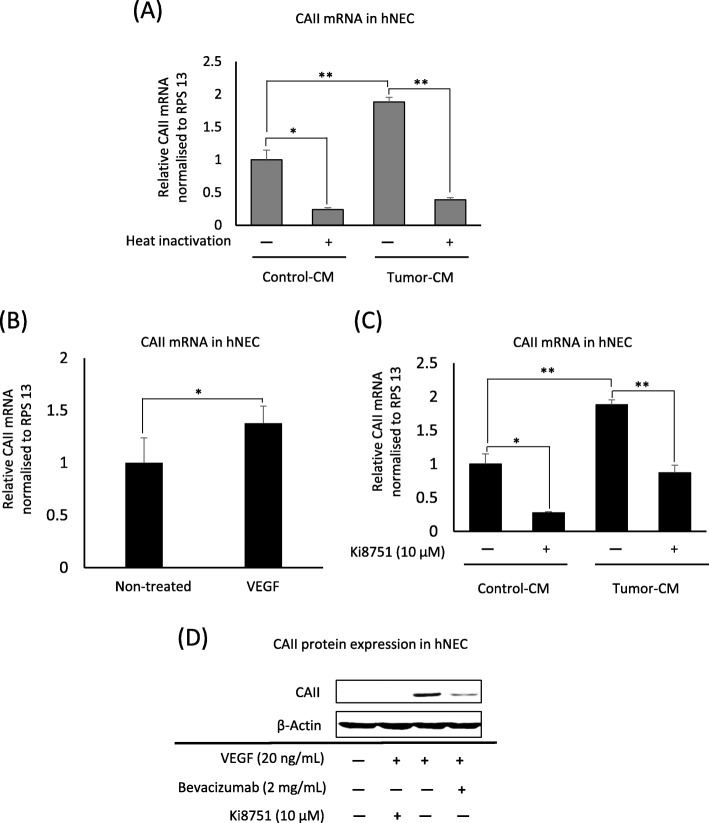
Tumor-derived factors induce CAII upregulation in endothelial cells. **a** Human NECs (hNECs) were treated with tumor or control-conditioned medium for 24 h. CAII mRNA expression was determined by RT-qPCR. **b** hNECs were stimulated with 20 ng/mL VEGF for 24 h, and RNA was isolated. CAII expression was determined by RT-qPCR. (C) hNECs were exposed to tumor-CM or control CM containing 10 μM Ki8751 (VEGFR2 inhibitor) for 24 h. CAII expression was determined by RT-qPCR. **d** hNECs were stimulated with 20 ng/mL VEGF alone or in combination with 10 μM Ki8751 (VEGFR2 inhibitor) or 2 mg/mL bevacizumab (VEGF neutralizing antibody). The cells were cultured for 36 h, and the protein was collected for analysis by western blotting. All data presented as mean ± SD, *P < 0.01, **P < 0.001, by two-tailed unpaired Student’s *t*-test, *n* = 3

Knowing that tumor-derived factors modulate the function of resident stromal cells in the tumor microenvironment, we used normal human NECs (hNECs) to investigate the contribution of tumor-derived substances released into the tumor-conditioned medium (tumor-CM) on CAII upregulation in TECs. Treating hNECs with tumor-CM for 24 h led to a significant upregulation of CAII mRNA expression, which was canceled upon tumor-CM heat inactivation (Fig. 5a). CAII expression was also slightly increased by hNEC conditioned-medium (control-CM) (Fig. 5a). The above results indicated that CAII upregulation in TECs might be induced by the proteins released from tumor cells, which may also be present in the EGM-2MV medium. Since our previous reports showed that A375-SM-CM contains significant amounts of vascular endothelial growth factor (VEGF) [6], we investigated the contribution of VEGF to CAII expression. VEGFA treatment increased CAII mRNA (Fig. 5b) and protein expressions in hNECs. VEGF acts through VEGF receptor tyrosine kinases, as indicated by VEGFR1, VEGFR2, and VEGFR3 [30]. Due to the high expression of VEGFR2/Kdr in TECs (Fig. 1c), we targeted the VEGFR2 in hNECs. The kinase inhibitor, Ki8751, selectively inhibited VEGFR2, and its introduction into tumor-CM decreased CAII mRNA expression in hNECs (Fig. 5c) and subsequently canceled the VEGFA-induced CAII protein expression (Fig. 5d). The VEGFA neutralizing antibody bevacizumab also reduced CAII protein expression in VEGFA-stimulated hNECs (Fig. 5d). Taken together, a new role for VEGFA and VEGFR2 in CAII upregulation in endothelial cells was shown in this study. This observation may explain the upregulated CAII expression in ECs obtained from tumor blood vessels.

### CAs’ pharmacological inhibition increased vessel maturity and decreased lung metastasis

**Fig. 6:**
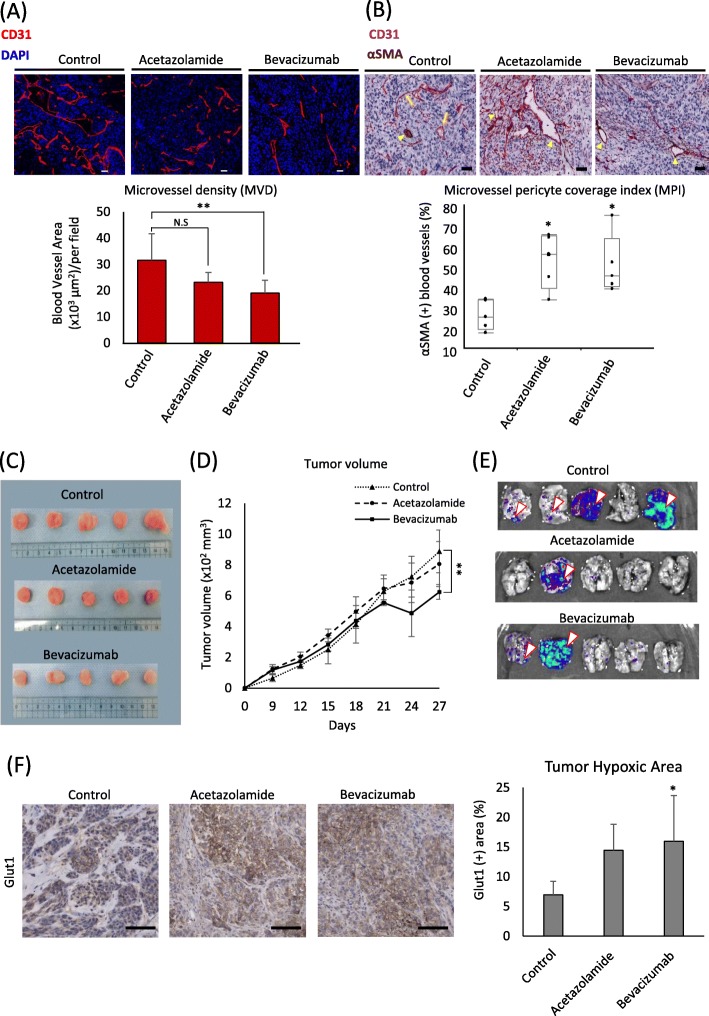
CAs’ pharmacological inhibition increased vessel maturity and decreased lung metastasis. **a** Tumor microvessel density (MVD) was analyzed by quantifying the CD31-positively stained area in tumor sections from each group by ImageJ. Blood vessel hotspots were selected (25 hotspots per treatment group). Representative images of tumor sections fixed and stained with anti-CD31 antibody (red) to identify the blood vessels and counterstained with DAPI (blue). Scale bar, 50 μm. **b** The microvessel pericyte coverage index (MPI) was analyzed by counting the vessels which stained positively for both CD31 and α-SMA (yellow arrowheads) among all CD31-positive vessels (yellow arrows) in blood vessel hotspots (15 hotspots per treatment group). Scale bar, 50 μm. **c** Images of whole tumors resected from the mice under each treatment condition after 27 days of drug treatment. **d** Tumor volume was calculated from the measurement obtained from the tumor-bearing mice using a pair of calipers. The measurements were taken on the indicated days. **e** Tumor metastasis was observed by detecting tumor cell luminescence intensity in the lungs (arrowhead) using IVIS spectrum. (F) Tumor hypoxia was measured by staining Glut1 expression in the tumor sections. The Glut1 staining was observed in the tumor cells. Scale bar, 100 μm. All data presented as mean ± SD, *P < 0.01, **P < 0.001 by ANOVA

Tumor-bearing mice were treated with acetazolamide, a carbonic anhydrase inhibitor, and bevacizumab, and their antiangiogenic effects were assessed comparatively. Bevacizumab significantly inhibited microvessel density (MVD) in the tumors more than acetazolamide (Fig. 6a). Since MVD is not the sole measure of vascular development and perivascular cells also play a role in vessel stability and maturation [31], we examined the perivascular cell coverage on the tumors’ blood vessels by calculating MPI. Surprisingly, we found that acetazolamide-treated tumors had a significantly higher MPI than non-treated tumors, which reached a comparable level to bevacizumab-treated tumors (Fig. 6b). The observed decrease in tumor angiogenesis was also evident through the pale coloration of the tumors in the bevacizumab-treatment group as compared to tumors in the control and acetazolamide-treatment groups (Fig. 6c). Furthermore, angiogenesis inhibition by bevacizumab led to a significant decrease in tumor size, whereas in the acetazolamide-treated group, the difference was not significant (Fig. 6d). Compared with the control group, lung metastasis was decreased in the treatment groups (Fig. 6e).

Similar to other antiangiogenic drugs, bevacizumab reportedly induces tumor hypoxia [32] through a decrease in blood vessel density, which can lead to epithelial-to-mesenchymal transition [33], tumor aggressiveness, and metastasis. We compared the tumors’ hypoxic area among the three groups by staining Glut1. In bevacizumab-treated tumors, the Glut1-positive area was significantly higher than in the control group but comparable to acetazolamide-treated tumors (Fig. 6f). Collectively, our findings show that CA inhibition by acetazolamide favors tumor blood vessel maturation via enhanced perivascular cell coverage and reduction in lung metastasis, with a nonsignificant decrease in tumor angiogenesis.

## Discussion

TECs support tumoral growth, contributing to cancer progression, without succumbing to harsh microenvironmental conditions, such as low pH/acidity, and excessive lactate released from tumor cells. We have demonstrated that TECs are well equipped to proliferate and survive in either lactic acidosis or nutrient-replete conditions, due to the upregulated expression of the pH regulator CAII. Our data shows that CAII expression in the tumor endothelium occurs partially as a consequence of tumor-derived VEGF and its signaling via VEGFR2. Additionally, CA inhibition exerts significant positive effects on blood vessel maturity in vivo.

Recent studies have demonstrated that, apart from tumor cells, cancer-associated fibroblasts undergo increased aerobic glycolysis and lactate production [34]. Similarly, the TECs used in this study were found to be highly glycolytic and to produce more lactate than NECs. Our findings are consistent with those of previous reports, which described that TECs isolated from B16-F10 melanoma and mouse ovarian epithelial papillary serous adenocarcinoma tumors developed in the liver were hyperglycolytic [14, 35]. Although in those studies the analyzed TECs had non-tumor NECs obtained from livers in the isolated TEC fraction, as opposed to our TECs, which were isolated from tumor tissue free from NEC contaminations, their results and ours show that TECs have altered their glucose metabolism.

Lactic acid sustains tumor cell survival under lactic acidosis [36], and Xie et al. showed that lactic acidosis supports the efficient use of glycolytic products to maintain 4 T1 proliferation [37]. In the present study, exposure of TECs to lactic acid may have driven the use of the glycolytic metabolite stores already present in TECs, thereby sustaining their proliferation. On the other hand, both the absence of abundant glycolytic metabolite stores and the acidity effects on NECs may explain the lack of significant increase in NEC numbers when exposed to lactic acid. Additionally, TECs may potentially possess the ability to use lactate as a metabolic substrate to support anabolism in a low pH environment, given their progressive growth with increasing concentrations of lactic acid. However, further studies are required to confirm this ability of TECs since this has not been previously observed in normal human ECs [20].

Vegran et al. demonstrated that siRNA silencing of endothelial cell MCT1 greatly impeded tumor microvascular network development in vivo [13]. This report may partly explain the effect of MCT1 siRNA on TECs observed in our study since TECs originated from tumors and may, therefore, require MCT1 expression to function well, which makes them more susceptible to MCT1 inhibition.

MCT transport activity within cells is supported by the CO_2_ hydrating CAs through non-catalytic interactions. The CAs function as proton antennae to facilitate the co-transport of proton/monocarboxylate by MCTs [26, 27]. In the present study, the upregulated expression of CAII and CAIII may imply that these cytosolic enzymes are significant for the function of TECs, unlike extracellular or membrane-bound CAs. CAII expression in the endothelium of our xenograft tumors and human renal cell carcinoma supports the upregulated expression of CAII mRNA and protein in the isolated TECs. Furthermore, the expression of CAII in tumor rather than normal blood vessels, as analyzed by IHC [17], further supports the present findings.

Among the CAs known to support MCT function, it was recently shown by Ames et al. (2018) that interfering with the proteoglycan domain of CAIX reduced not only proton-coupled lactate transport but also hypoxic breast cancer cell proliferation and migration [27]. This observation suggested that the contribution of CAIX to MCT activity subsequently affected cell survival. In the present study, CAII inhibition by siRNA led to a similar phenotypic outcome in TECs, regardless of the metabolic substrate or acidity. The inhibitory effects of CAII knockdown on TEC proliferation indicate that CAII is essential for TEC proangiogenic functions, such as proliferation. Moreover, identification of the CAII subunit responsible for regulating endothelial cell proliferation will shed light on its role in angiogenesis.

MCT and CA inhibition strategies have individually yielded promising results in cancer treatment [38, 39]. Although in the present study we did not inhibit MCT1 in vivo, targeting MCT1 and CAII together may synergistically increase the therapeutic benefits of their inhibitors through decreasing cancer cell proliferation and inhibiting tumor angiogenesis, which supports tumor growth and metastasis.

Apart from CAII and MCT interactions, CAII can also enhance the proton extrusion activity of other pH regulating proteins, such as NHE1 [40]. CAII was postulated to be involved in endothelial cell proliferation via its pH regulatory role in proliferating and differentiating endothelial cells in the microvessels of the developing human brain [41]. Similarly, CAII may perform a pH regulatory role in lactic acidosis, by activating NHE1 to increase H^+^ extrusion.

Furthermore, CAII may contribute to cellular metabolism by working together with mitochondrial and extracellular CAs to produce HCO_3_^−^ required for biosynthetic pathways such as gluconeogenesis or lipogenesis [42]. Wei et al. showed that lactate induces the expression of the gluconeogenesis enzyme phosphoenolpyruvate carboxykinase in THP-1 monocytes to produce glucose which enhanced tumor growth [43]; similarly the higher levels of lactate that TECs are exposed to in the tumor microenvironment, and the scarcity of glucose intratumorally, could lead to such changes in TECs. A report indicating that co-culturing lymphatic endothelial cells with breast cancer cell lines enriched the gluconeogenesis pathway in the co-cultured endothelial cells as compared to the controls, further supports this suggestion [44]. Additionally, in both NECs and TECs, HCO_3_^−^ will combine with acetyl-CoA in the carboxylation reaction catalyzed by acetyl CoA carboxylase to form malonyl Co-A, as the initial step of lipogenesis. EC lipogenesis is necessary for EC migration and filopodia formation [45] and in TECs may enhance tumor angiogenesis. However, more studies are required to prove the contribution of CAII to these biosynthetic processes in the ECs.

To the best of our knowledge, no previous reports have investigated the role of VEGF/VEGFR2 signaling in CAII expression or function. However, using bevacizumab and VEGFR2 kinase inhibitor, we have demonstrated that VEGF and its activation via VEGFR2 are essential for CAII expression in endothelial cells. A previous study showed that CAII mRNA expression in HUVECs was upregulated by acidity and hypoxia [17]. Even though the mechanism was not elucidated, VEGF may be involved since both acidity [46, 47] and hypoxia [48] induce VEGF production. Collectively, we can suggest that various tumor microenvironmental conditions, including VEGF, may contribute to CAII induction in TECs, and this will make TECs well equipped for the harsh conditions in the tumor microenvironment.

Current targets of tumor angiogenesis have not successfully eliminated cancer or effectively decreased angiogenesis due to various mechanisms of acquired resistance or side effects caused by nonspecific effects on NECs [49, 50]. Developing novel drugs is, therefore required. In the present study, although in vivo treatment with acetazolamide showed an antimetastatic effect, its antiangiogenic effect was not significant. Considering these results and as previously observed for adenocarcinoma tumors [23], acetazolamide alone is not enough for use as an antiangiogenic drug to a similar extent as bevacizumab. However, it can be used in combination with conventional antiangiogenic drugs to improve antiangiogenic therapy by enhancing the normalization of tumor blood vessels through improved pericyte coverage. Perivascular cells, such as pericytes, support the establishment of matured blood vessels by physically nteracting with the newly formed tumor vessels or by secreting growth factors to support the ECs [51, 52]. Upon pericyte attachment, blood vessels become more mature and stabilized. The vessel maturation may further prevent the invasion of tumor cells into the blood vessels and subsequent escape through the vasculature to metastatic organs [53] or even improve drug delivery to the tumor cells [54].

## Conclusion

We have shown that TEC proliferation is not inhibited by tumor metabolic end-products like lactic acid due to the expression of CAII in TEC, unlike normal endothelial cells. Furthermore, VEGF/VEGFR2 signaling pathway is involved in the upregulation of CAII in TECs. Therefore, pH regulators like CAII may be good therapeutic targets to enhance the efficacy of antiangiogenic drugs that target VEGF or its receptor.

## Supplementary information

**Additional file 1: Table S1.** List of primary and secondary antibodies used in flow cytometry, immunohistochemistry and immunoblotting.

**Additional file 2: Table S2.** List of primers for PCR analysis.

**Additional file 3: Figure S1.** Gene expression of some cancer- associated pH regulators. (A) NHE1 mRNA expression in TECs and NECs was evaluated by RT-qPCR. (B) mRNA expression of the pH regulators Gpr4 and Gpr65 evaluated by RT-qPCR in TECs and NECs. All data is presented as mean ± SD; N.S., not significant, by two-tailed unpaired Student’s *t*-test, *n* = 3.

## Data Availability

Data sharing is not applicable to this article as no datasets were generated or analyzed during the current study.

## Abbreviations

CA: Carbonic anhydrase
CM: Conditioned medium
DMEM: Dulbecco’s Minimum Essential Medium
DT: Diphtheria toxin
ECGS: Endothelial cell growth supplement
ELISA: Enzyme-linked immunosorbent assay
FBS: Fetal bovine serum
hNEC: Human normal endothelial cells
IHC: Immunohistochemistry
MCT: Monocarboxylate transporter
MEM: Minimum Essential Medium
MPI: Microvessel pericyte coverage index
MVD: Microvessel density
NEC: Normal endothelial cells
NHE1: Sodium/hydrogen exchanger 1
RCC: Renal cell carcinoma
TEC: Tumor endothelial cells
VEGF: Vascular endothelial growth factor
VEGFR: Vascular endothelial growth factor receptor
αSMA: Alpha smooth muscle actin

## References

[1] Verheul HM, Voest EE, Schlingemann RO (2004). Are tumours angiogenesis-dependent?. J Pathol.

[2] Hida K, Hida Y, Amin DN, Flint AF, Panigrahy D, Morton CC (2004). Tumor-associated endothelial cells with cytogenetic abnormalities. Cancer Res.

[3] Ohga N, Ishikawa S, Maishi N, Akiyama K, Hida Y, Kawamoto T (2012). Heterogeneity of tumor endothelial cells: comparison between tumor endothelial cells isolated from high- and low-metastatic tumors. Am J Pathol.

[4] Maishi N, Ohba Y, Akiyama K, Ohga N, Hamada J, Nagao-Kitamoto H (2016). Tumour endothelial cells in high metastatic tumours promote metastasis via epigenetic dysregulation of biglycan. Sci Rep.

[5] Hellebrekers DMEI, Castermans K, Viré E, Dings RPM, Hoebers NTH, Mayo KH (2006). Epigenetic regulation of tumor endothelial cell Anergy: silencing of intercellular adhesion Molecule-1 by histone modifications. Cancer Res.

[6] Akiyama K, Ohga N, Hida Y, Kawamoto T, Sadamoto Y, Ishikawa S (2012). Tumor endothelial cells acquire drug resistance by MDR1 up-regulation via VEGF signaling in tumor microenvironment. Am J Pathol.

[7] Osawa T, Ohga N, Akiyama K, Hida Y, Kitayama K, Kawamoto T (2013). Lysyl oxidase secreted by tumour endothelial cells promotes angiogenesis and metastasis. Br J Cancer.

[8] Hojo T, Maishi N, Towfik AM, Akiyama K, Ohga N, Shindoh M (2017). ROS enhance angiogenic properties via regulation of NRF2 in tumor endothelial cells. Oncotarget..

[9] Vander Heiden MG, Cantley LC, Thompson CB (2009). Understanding the Warburg effect: the metabolic requirements of cell proliferation. Science (New York, NY).

[10] Colegio OR, Chu NQ, Szabo AL, Chu T, Rhebergen AM, Jairam V (2014). Functional polarization of tumour-associated macrophages by tumour-derived lactic acid. Nature..

[11] Fischer K, Hoffmann P, Voelkl S, Meidenbauer N, Ammer J, Edinger M (2007). Inhibitory effect of tumor cell-derived lactic acid on human T cells. Blood..

[12] Burbridge MF, West DC, Atassi G, Tucker GC (1999). The effect of extracellular pH on angiogenesis in vitro. Angiogenesis..

[13] Vegran F, Boidot R, Michiels C, Sonveaux P, Feron O (2011). Lactate influx through the endothelial cell monocarboxylate transporter MCT1 supports an NF-kappaB/IL-8 pathway that drives tumor angiogenesis. Cancer Res.

[14] Cantelmo AR, Conradi LC, Brajic A, Goveia J, Kalucka J, Pircher A (2016). Inhibition of the glycolytic activator PFKFB3 in endothelium induces tumor vessel normalization, impairs metastasis, and improves chemotherapy. Cancer Cell.

[15] Doherty JR, Cleveland JL (2013). Targeting lactate metabolism for cancer therapeutics. J Clin Invest.

[16] Damaghi M, Wojtkowiak JW, Gillies RJ (2013). pH sensing and regulation in cancer. Front Physiol.

[17] Yoshiura K, Nakaoka T, Nishishita T, Sato K, Yamamoto A, Shimada S (2005). Carbonic anhydrase II is a tumor vessel endothelium-associated antigen targeted by dendritic cell therapy. Clin Cancer Res.

[18] Haapasalo J, Nordfors K, Jarvela S, Bragge H, Rantala I, Parkkila AK (2007). Carbonic anhydrase II in the endothelium of glial tumors: a potential target for therapy. Neuro-oncology..

[19] Korhonen K, Parkkila AK, Helen P, Valimaki R, Pastorekova S, Pastorek J (2009). Carbonic anhydrases in meningiomas: association of endothelial carbonic anhydrase II with aggressive tumor features. J Neurosurg.

[20] Sonveaux P, Copetti T, De Saedeleer CJ, Vegran F, Verrax J, Kennedy KM (2012). Targeting the lactate transporter MCT1 in endothelial cells inhibits lactate-induced HIF-1 activation and tumor angiogenesis. PLoS One.

[21] Hirayama A, Kami K, Sugimoto M, Sugawara M, Toki N, Onozuka H (2009). Quantitative metabolome profiling of colon and stomach cancer microenvironment by capillary electrophoresis time-of-flight mass spectrometry. Cancer Res.

[22] Ohga N, Hida K, Hida Y, Muraki C, Tsuchiya K, Matsuda K (2009). Inhibitory effects of epigallocatechin-3 gallate, a polyphenol in green tea, on tumor-associated endothelial cells and endothelial progenitor cells. Cancer Sci.

[23] Faes S, Uldry E, Planche A, Santoro T, Pythoud C, Demartines N (2016). Acidic pH reduces VEGF-mediated endothelial cell responses by downregulation of VEGFR-2; relevance for anti-angiogenic therapies. Oncotarget..

[24] Halestrap AP, Price NT (1999). The proton-linked monocarboxylate transporter (MCT) family: structure, function and regulation. Biochem J.

[25] Sonveaux P, Vegran F, Schroeder T, Wergin MC, Verrax J, Rabbani ZN (2008). Targeting lactate-fueled respiration selectively kills hypoxic tumor cells in mice. J Clin Invest.

[26] Noor SI, Jamali S, Ames S, Langer S, Deitmer JW, Becker HM. A surface proton antenna in carbonic anhydrase II supports lactate transport in cancer cells. Elife. 2018;7. 10.7554/eLife.35176 PMC5986270 29809145

[27] Ames S, Pastorekova S, Becker HM (2018). The proteoglycan-like domain of carbonic anhydrase IX mediates non-catalytic facilitation of lactate transport in cancer cells. Oncotarget..

[28] Zhou Y, Mokhtari RB, Pan J, Cutz E, Yeger H (2015). Carbonic anhydrase II mediates malignant behavior of pulmonary neuroendocrine tumors. Am J Respir Cell Mol Biol.

[29] Parkkila AK, Herva R, Parkkila S, Rajaniemi H (1995). Immunohistochemical demonstration of human carbonic anhydrase isoenzyme II in brain tumours. Histochem J.

[30] Stuttfeld E, Ballmer-Hofer K (2009). Structure and function of VEGF receptors. IUBMB Life.

[31] Fakhrejahani E, Toi M (2012). Tumor angiogenesis: pericytes and maturation are not to be ignored. J Oncol.

[32] Vaeteewoottacharn K, Kariya R, Dana P, Fujikawa S, Matsuda K, Ohkuma K (2016). Inhibition of carbonic anhydrase potentiates bevacizumab treatment in cholangiocarcinoma. Tumour Biol.

[33] Iwadate Y (2016). Epithelial-mesenchymal transition in glioblastoma progression. Oncol Lett.

[34] Zhang D, Wang Y, Shi Z, Liu J, Sun P, Hou X (2015). Metabolic reprogramming of cancer-associated fibroblasts by IDH3alpha downregulation. Cell Rep.

[35] Zhang L, Li S, Li L, Chen Z, Yang Y (2018). COX2 inhibition in the endothelium induces glucose metabolism normalization and impairs tumor progression. Mol Med Rep.

[36] Wu H, Ding Z, Hu D, Sun F, Dai C, Xie J (2012). Central role of lactic acidosis in cancer cell resistance to glucose deprivation-induced cell death. J Pathol.

[37] Xie J, Wu H, Dai C, Pan Q, Ding Z, Hu D (2014). Beyond Warburg effect--dual metabolic nature of cancer cells. Sci Rep.

[38] Benjamin D, Robay D, Hindupur SK, Pohlmann J, Colombi M, El-Shemerly MY (2018). Dual Inhibition of the Lactate Transporters MCT1 and MCT4 Is Synthetic Lethal with Metformin due to NAD+ Depletion in Cancer Cells. Cell Rep.

[39] Abdel Gawad NM, Amin NH, Elsaadi MT, Mohamed FMM, Angeli A, De Luca V (2016). Synthesis of 4-(thiazol-2-ylamino)-benzenesulfonamides with carbonic anhydrase I, II and IX inhibitory activity and cytotoxic effects against breast cancer cell lines. Bioorg Med Chem.

[40] Li X, Alvarez B, Casey JR, Reithmeier RA, Fliegel L (2002). Carbonic anhydrase II binds to and enhances activity of the Na+/H+ exchanger. J Biol Chem.

[41] Kida E, Palminiello S, Golabek AA, Walus M, Wierzba-Bobrowicz T, Rabe A (2006). Carbonic anhydrase II in the developing and adult human brain. J Neuropathol Exp Neurol.

[42] Supuran Claudiu (2018). Carbonic Anhydrases and Metabolism. Metabolites.

[43] Wei L, Zhou Y, Yao J, Qiao C, Ni T, Guo R (2015). Lactate promotes PGE2 synthesis and gluconeogenesis in monocytes to benefit the growth of inflammation-associated colorectal tumor. Oncotarget..

[44] Acevedo-Acevedo S, Millar DC, Palecek SP (2018). Abstract 3481: elucidating the metabolic crosstalk between lymphatic endothelial cells and breast cancer using 1H NMR metabolomics. Cancer Res.

[45] Glatzel DK, Koeberle A, Pein H, Loser K, Stark A, Keksel N (2018). Acetyl-CoA carboxylase 1 regulates endothelial cell migration by shifting the phospholipid composition. J Lipid Res.

[46] Xu L, Fukumura D, Jain RK (2002). Acidic extracellular pH induces vascular endothelial growth factor (VEGF) in human glioblastoma cells via ERK1/2 MAPK signaling pathway: mechanism of low pH-induced VEGF. J Biol Chem.

[47] D'Arcangelo D, Facchiano F, Barlucchi LM, Melillo G, Illi B, Testolin L (2000). Acidosis inhibits endothelial cell apoptosis and function and induces basic fibroblast growth factor and vascular endothelial growth factor expression. Circ Res.

[48] Namiki A, Brogi E, Kearney M, Kim EA, Wu T, Couffinhal T (1995). Hypoxia induces vascular endothelial growth factor in cultured human endothelial cells. J Biol Chem.

[49] Zarrin B, Zarifi F, Vaseghi G, Javanmard SH (2017). Acquired tumor resistance to antiangiogenic therapy: mechanisms at a glance. J Res Med Sci.

[50] Bergers G, Hanahan D (2008). Modes of resistance to anti-angiogenic therapy. Nat Rev Cancer.

[51] Darland DC, Massingham LJ, Smith SR, Piek E, Saint-Geniez M, D'Amore PA (2003). Pericyte production of cell-associated VEGF is differentiation-dependent and is associated with endothelial survival. Dev Biol.

[52] Raza A, Franklin MJ, Dudek AZ (2010). Pericytes and vessel maturation during tumor angiogenesis and metastasis. Am J Hematol.

[53] Welén K, Jennbacken K, Tesan T, Damber JE (2008). Pericyte coverage decreases invasion of tumour cells into blood vessels in prostate cancer xenografts. Prostate Cancer Prostatic Dis.

[54] Kim SJ, Jung KH, Son MK, Park JH, Yan HH, Fang Z (2017). Tumor vessel normalization by the PI3K inhibitor HS-173 enhances drug delivery. Cancer Lett.

