# Supplementary material for: Carbonic anhydrase 2 (CAII) supports tumor blood endothelial cell survival under lactic acidosis in the tumor microenvironment
Source: Cell Commun Signal. 2019 Dec 17;17:169. doi: 10.1186/s12964-019-0478-4 (PMC6918655; doi:10.1186/s12964-019-0478-4)
Supplement: Supplementary file 2 — Additional file 1: Table S1. List of primary and secondary antibodies used in flow cytometry, immunohistochemistry and immunoblotting. [file 12964_2019_478_MOESM2_ESM.docx]

Supplementary Table S1. List of primary and secondary antibodies used in flow cytometry, immunohistochemistry and immunoblotting.

|  | Antibody | Application/Dilution | Catalogue No | Clone | Supplier |
| --- | --- | --- | --- | --- | --- |
| 1 | Alexa Fluor 647-conjugated anti-mouse CD31 | FC/ 1:100 | 102416 | 390 | Biolegend |
| 2 | FITC-conjugated anti-mouse CD45 | FC/ 1:100 | 103108 | 30-F11 | BioLegend, |
| 3 | Rat anti-mouse CD31 | IHC-Fr/ 1:400 | 553370 | MEC 13.3 | BD Pharmingen |
| 4 | Rabbit anti-CAII | IHC-Fr/ 1:100 | ab191343 | polyclonal | abcam |
| 5 | Mouse anti-MCT1 | IB/ 1:1000 | ab90582 | polyclonal | abcam |
| 6 | Rabbit anti-CD31 | IHC-P/ 1:400 | ab28364 | polyclonal | abcam |
| 7 | Rabbit anti-CAII | IB/ 1:1000 | ab124687 | EPR5195 | abcam |
| 8 | Rabbit anti-β-actin | IB/ 1:3000 | #4970 | 13E5 | Cell Signaling Technology |
| 9 | Rabbit anti-alpha smooth muscle actin | IHC-P/ 1:200 | ab5694 | polyclonal | abcam |
| 10 | Rabbit anti-Glut1 | IHC-P/ 1:1000 | ab115730 | EPR3915 | abcam |
| 11 | HRP-linked Goat anti-rabbit IgG | IHC-P/ 1:100 | P0448 | polyclonal | DAKO |
| 12 | AP-linked Goat anti-rabbit IgG | IHC-P/ 1:50 | D0487 | polyclonal | DAKO |

FC: flow cytometry, IHC: Immunohistochemistry, IB: Immunoblotting
