# Supplementary material for: Carbonic anhydrase 2 (CAII) supports tumor blood endothelial cell survival under lactic acidosis in the tumor microenvironment
Source: Cell Commun Signal. 2019 Dec 17;17:169. doi: 10.1186/s12964-019-0478-4 (PMC6918655; doi:10.1186/s12964-019-0478-4)
Supplement: Supplementary file 3 — Additional file 2: Table S2. List of primers for PCR analysis. [file 12964_2019_478_MOESM3_ESM.docx]

**Supplementary Table S2. List of primers for PCR analysis**

| Gene name | Sequence |
| --- | --- |
| *Mouse Rps13* | *forward* 5′-TGCGGCTTGATTTCCTGTGCCG-3′ |
|  | *reverse* 5′-GCGTGCATGCGACCCATGATGATG-3′ |
| *Mouse Cdh5* | *forward* 5′-TGCTCACGGACAAGATCAGCTC-3′ |
|  | *reverse* 5′-GGAAAGTTAGGGCCTGCCATTG-3′ |
| *Mouse Eng* | *forward* 5′-CTTCCAAGGACAGCCAAGAG-3′ |
|  | *reverse* 5′-GGGTCATCCAGTGCTGCTAT-3′ |
| *Mouse Pecam1* | *forward* 5′-TCGACCCTAAGAACGGAAGGC-3′ |
|  | *reverse* 5′-CTCTTCTCGGGACATGGACGAC-3′ |
| *Mouse Icam1* | *forward* 5′-AcGAAGCTTCTTTTGCTCTGCC-3′ |
|  | *reverse* 5′-GAGTCTGCTGAGACCCCTCTTG-3′ |
| *Mouse Flt1* | *forward* 5′-GAGGTAGTGCTAGTGGTGGTGG-3′ |
|  | *reverse* 5′-TCCCCTCCTGCTTCTGCTTG-3′ |
| *Mouse Kdr* | *forward* 5′-GCCCTGCTGTGGTCTCACTAC-3′ |
|  | *reverse* 5′-CAAAGCATTGCCCATTCGAT-3′ |
| *Mouse Ptprc* | *forward 5*′-CCTCAAACTTCGACGGAGAG-3′ |
|  | *reverse* 5′-CACTTGCACCATCAGACACC-3′ |
| *Mouse Itgam* | *forward* 5′-CCAAGAAAGTAGCAAGGAGTGTG-3′ |
|  | *reverse* 5′-AGGGTCTAAGCCAGGTCATAAG-3′ |
| *Mouse Slc16a1* | *forward* 5′-ACACCAAGTGGATCAGACCTCG-3′ |
|  | *reverse* 5′-GGTTGTAGACAAAGGGGCAAGC-3′ |
| *Mouse Car2* | *forward* 5′-TGCGTCCAAGAGCATTGTCAAC-3′ |
|  | *reverse* 5′-GTCACTGAGGGGTCCTCCTTTC-3′ |
| *Mouse Car3* | *forward 5'- CTCTTCGGGCAAGAAACTCTGC -3'* |
|  | *reverse 5'- GGTTGCATGTGACTGCTTCTCC -3'* |
| *Mouse Car4* | *forward* 5′-TTGGTGATTGACCCTAGGCTGG-3′ |
|  | *reverse* 5′-AGTCTGGGGTTCACCTTTGTCC-3′ |
| *Mouse Car9* | *forward* 5′-TTCCTGCTGCTCCAAGTGTCTG-3′ |
|  | *reverse* 5′-TCAGGTGCATCCTCTTCACTGG-3′ |
| *Mouse Slc9a1* | *forward* 5′-TGGTGAACGAGGAGTTGAAGGG-3′ |
|  | *reverse* 5′-ACTTGATCCAGGGGTGAAGACG-3′ |
| *Mouse Gpr4* | *forward* 5′-AACTGTCATCCTGCACCCTTCC-3′ |
|  | *reverse* 5′-CGCCCATGATGACAAACTCCTG-3′ |
| *Mouse Gpr65* | *forward* 5′-ATGCGTATCCTTTCTGCAAGCG-3′ |
|  | *reverse* 5′-ACTGCTAAATAGCGGTCCAGGG-3′ |
| *Human RPS13* | *forward* 5′-TCTCCTTTCGTTGCCTGATCGCC-3′ |
|  | *reverse* 5′-ACTTCAACCAAGTGGGGACGCTGC-3′ |
| *Human CAR2* | *forward* 5′-TCCCCTGTTGACATCGACACTC-3′ |
|  | *reverse* 5′-ACCAAGTGAAGCTGCTTTGTCC-3′ |
