# Supplementary figures and images for: Carbonic anhydrase 2 (CAII) supports tumor blood endothelial cell survival under lactic acidosis in the tumor microenvironment
Source: Cell Commun Signal. 2019 Dec 17;17:169. doi: 10.1186/s12964-019-0478-4 (PMC6918655; doi:10.1186/s12964-019-0478-4)

(A)

## Sodium–hydrogen antiporter

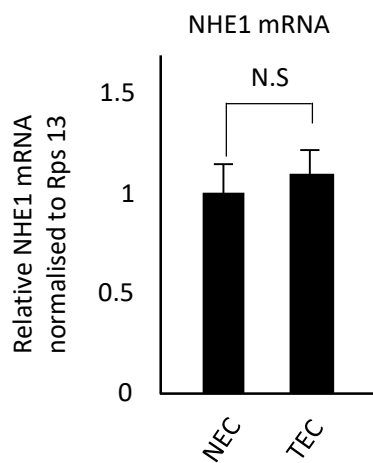

(B)

## Proton-sensing G protein-coupled receptors

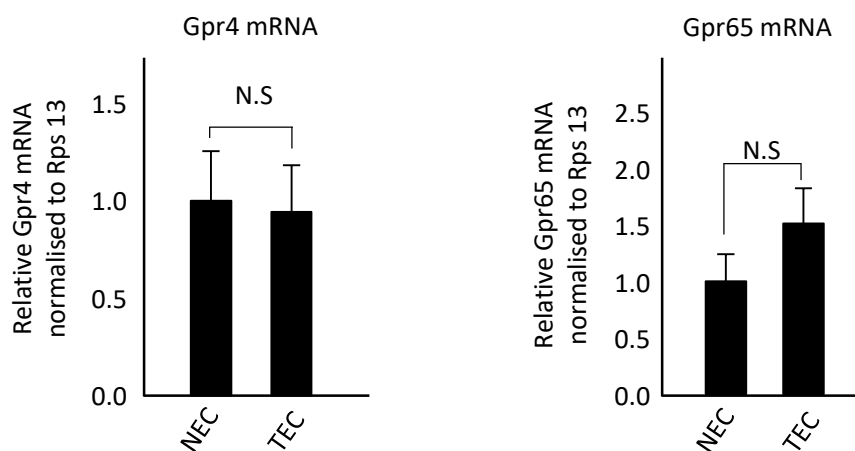

Supplement: Supplementary file 4 — Additional file 3: Figure S1. Gene expression of some cancer- associated pH regulators. (A) NHE1 mRNA expression in TECs and NECs was evaluated by RT-qPCR. (B) mRNA expression of the pH regulators Gpr4 and Gpr65 evaluated by RT-qPCR in TECs and NECs. All data is presented as mean ± SD; N.S., not significant, by two-tailed unpaired Student’s t-test, n = 3. [file 12964_2019_478_MOESM4_ESM.pdf]
